# Whisker encoding of mechanical events during active tactile exploration

**DOI:** 10.3389/fnbeh.2012.00074

**Published:** 2012-11-06

**Authors:** Yves Boubenec, Daniel E. Shulz, Georges Debrégeas

**Affiliations:** ^1^Unité de Neurosciences Information et Complexité, UPR 3293, Centre National de la Recherche ScientifiqueGif-sur-Yvette, France; ^2^FRE3231 Laboratoire Jean Perrin, Centre National de la Recherche Scientifique, UPMC Université Paris 6Paris, France

**Keywords:** exploration, rat, resonance, tactile, vibration, vibrissae, whiskers, whisking

## Abstract

Rats use their whiskers to extract a wealth of information about their immediate environment, such as the shape, position or texture of an object. The information is conveyed to mechanoreceptors located within the whisker follicle in the form of a sequence of whisker deflections induced by the whisker/object contact interaction. How the whiskers filter and shape the mechanical information and effectively participate in the coding of tactile features remains an open question to date. In the present article, a biomechanical model was developed that provides predictions of the whisker dynamics during active tactile exploration, amenable to quantitative experimental comparison. This model is based on a decomposition of the whisker profile into a slow, quasi-static sequence and rapid resonant small-scale vibrations. It was applied to the typical situation of a rat actively whisking across a solid object. Having derived the quasi-static sequence of whisker deformation, the resonant properties of the whisker were analyzed, taking into account the boundary conditions imposed by the whisker/surface contact. We then focused on two elementary mechanical events that are expected to trigger significant neural responses, namely (1) the whisker/object first contact and (2) the whisker detachment from the object. Both events were found to trigger a deflection wave propagating upward to the mystacial pad at constant velocity of ≈3–5 m/s. This yielded a characteristic mechanical signature at the whisker base, in the form of a large peak of negative curvature occurring ≈4 ms after the event has been triggered. The dependence in amplitude and lag of this mechanical signal with the main contextual parameters (such as radial or angular distance) was investigated. The model was validated experimentally by comparing its predictions to high-speed video recordings of shock-induced whisker deflections performed on anesthetized rats. The consequences of these results on possible tactile encoding schemes are briefly discussed.

## Introduction

The vibrissal system of the rat is one of the prominent model systems for investigating the mechanisms of sensory information processing in the tactile modality. Rats use their whiskers to sense their close environment and gather information about object features such as location (Krupa and Nicolelis, 2001; O'Connor et al., 2010a), shape (Brecht and Merzenich, 1997; Polley et al., 2005), texture (Carvell and Simons, 1990; Morita et al., 2011), and size (Anjum et al., 2006). Active movements of body, head (Milani et al., 1989; Carvell and Simons, 1990; Towal and Hartmann, 2006; Mitchinson et al., 2007) or whiskers themselves during whisking (Welker, 1964; Carvell and Simons, 1990; Berg and Kleinfeld, 2003) induce contact between the whisker and the probed environment. This mechanical interaction elicits sequences of whisker deflection. Each whisker is embedded in a follicle in the skin (Ebara et al., 2002), where mechanoreceptors transduce whisker base deflections into neural signals (Lichtenstein et al., 1990; Szwed et al., 2006). Neurons along the trigeminal pathway respond to various aspects of the whisker base movements, such as high acceleration events (Jadhav et al., 2009; Lottem and Azouz, 2009; Jadhav and Feldman, 2010), whisker speed (Arabzadeh et al., 2004), average noise level (Arabzadeh, 2005), or characteristic features of the whisker motion spectra (Hipp et al., 2006). These different properties of the whisker base dynamics are then processed by the central nervous systems to extract relevant features of the environment. In order to decipher the underlying neural code, one needs to relate the dynamics of the whisker to the physical and geometrical characteristics of the contacting surface. This amounts to understanding the way the whisker carries and shapes information from the contact point to the follicle.

**Figure 1:**
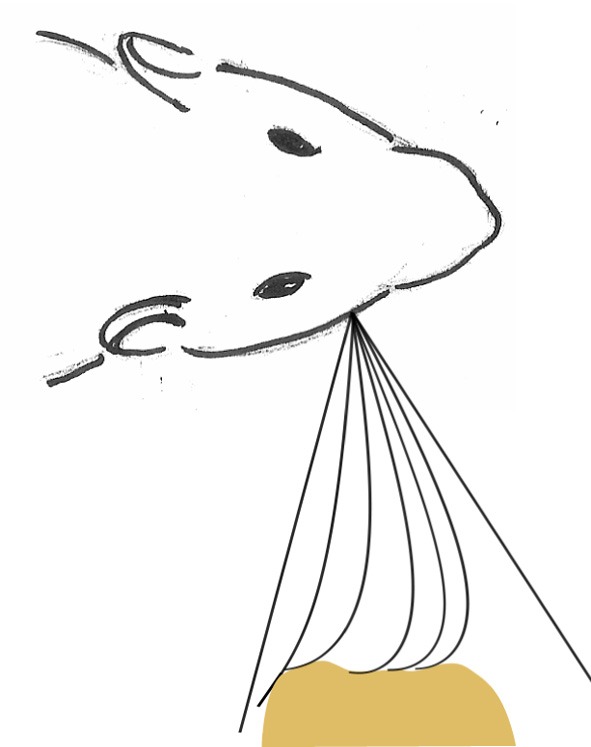
**Schematic view of a tactile exploration task**.

Consider a typical exploration task during which a rat whisks across an object (Figure 1). This sequence can be broken up into three consecutive phases. First, the whisker rotates freely in air; second, it slides over the object and is submitted to a frictional contact at the whisker tip; the whisker then detaches and pursues its motion in air (no contact). Recently, several groups have studied how the rat could extract the contour of an object from the time evolution of the torque at the base of each of its whiskers (Kaneko et al., 1998; Scholz and Rahn, 2004; Clements and Rahn, 2006; Kim and Möller, 2007; Solomon and Hartmann, 2010). The encoding mechanism requires proprioceptive information (the angular position of the whisker with respect to the snout) and the knowledge of the relationship between the radial distance and the resulting torque in the follicle. This relationship was derived by computing the successive equilibrium profiles of the whiskers for a non-frictional contact. Due to the slender geometry of the whisker, any change in the contact configuration (during the first contact or following the whisker detachment from the object) is expected to trigger a burst of whisker oscillations ignored in these quasi-static descriptions. During the sliding phase itself, stick-slip instabilities are bound to occur, which should also result in brief vibrating episodes. Several studies have suggested that these mechanical events are encoded by specific mechanoreceptors. In particular, Szwed et al. (2003) established that distinct populations of trigeminal ganglion neurons specifically respond to first contact and/or detachment. Other works (Ritt, 2008; Wolfe et al., 2008; Jadhav et al., 2009; Lottem and Azouz, 2009) have further suggested that surface roughness may in large part be encoded by the rate of discrete high acceleration events elicited by stick-slip instabilities at the whisker/object contact. Notice that analogous mechanical events, separating different action phases, have been shown to play a central role in the planning and control of manipulation tasks in the context of active human touch (Johansson et al., 2009).

In the present article, we focused on the encoding of the first contact and detachment events by the whisker. A biomechanical model is developed to quantitatively predict the sequence of whisker deformation that these events elicit in realistic conditions of tactile exploration. Specific predictions of the model were validated through high-speed video recordings of shock-induced whisker deflections. By investigating how various contextual parameters (such as radial and angular distance or friction coefficients) control the mechanical signature at the whisker base, we aimed to understand what information can be extracted by the neural system. The consequence of these results on possible tactile encoding schemes are discussed, which we believe may be of interest to the fields of whisker tactile perception as well as neurorobotics.

## Results

Our biomechanical approach is based on a decomposition of the whisker dynamics into rapid small amplitude resonant oscillations superimposed onto a slow (quasi-static) sequence of deformation. The oscillating term is further decomposed along a series of resonant modes whose spatial and temporal properties are computed numerically.

In a first section, we describe the quasi-static evolution of the whisker as it is swept across a rectangular obstacle. In the second section, the mode decomposition scheme used to describe the rapid dynamics of the whisker is presented. Two distinct mechanical events are then successively examined: (1) the initial contact (shock) between the whisker and the object; (2) the detachment of the whisker from the object. In both cases, the precise time-sequence of the whisker dynamics can be accurately predicted. A particular focus is put on the time-evolution of the moment at the whisker base (in the follicle) as it constitutes the relevant peripheral input for the mechanoreceptors.

### Quasi-static evolution of a whisker scanned across a rectangular object

**Figure 2:**
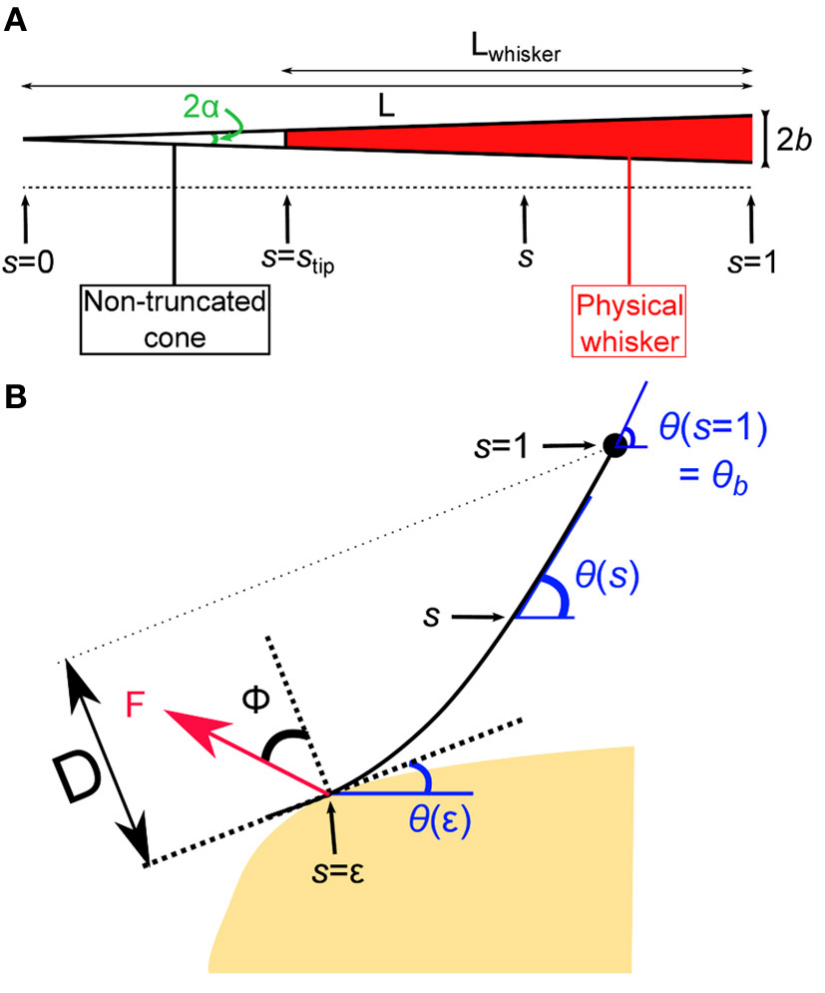
**Geometry of the whisker in contact. (A)** Whisker as a truncated cone. **(B)** The whisker is submitted to a localized frictional contact imposed at *s* = ε and oriented at a friction angle ϕ with respect to the direction normal to the surface. Notice that the whisker is locally tangent to the surface. The whisker rotates at constant rate around a fixed point that corresponds to the whisker base (*s* = 1).

In this first section, the quasi-static evolution of the whisker, i.e., the series of equilibrium configurations, is calculated as it is swept across a rectangular object. Several robotics studies analyzed the shape of a whisker submitted to a contact force. Most of those works used numerical solutions to determine whisker profiles (Scholz and Rahn, 2004; Clements and Rahn, 2006; Solomon and Hartmann, 2010). In the limit of small deflections, a small angle approximation can be used which yields an analytical solution to this mechanical problem (Birdwell et al., 2007). A large majority of those works, however, ignored any frictional interaction between the whisker and the substrate in their theoretical derivations. Nevertheless a few studies performed with artificial whiskers investigated the influence of frictional interactions for predicting radial distance (Solomon and Hartmann, 2008) and local object shape (Schroeder and Hartmann, 2012) during whisker touch. Here, we numerically derive the quasi-static sequence of whisker deflection as it is swept across an obstacle, taking into account the frictional force. In line with physiological observations made in rats (Voges et al., 2012) and other whisker-bearing animals (Williams and Kramer, 2010), the whisker is modeled as a truncated tapered rod. We denote *L* the length of the non-truncated cone, *b* the maximum (base) radius and α = *b*/*L* the cone angle (Figure 2A). In order to simplify the equations governing the whisker mechanics, the whisker profiles are described using a curvilinear coordinate *s* defined as the normalized arc length such that the cone tip (the end of the non-truncated whisker) position defines the origin *s* = 0 and the whisker base is located at *s* = 1. The radius of the whisker at position *s* thus reads *r*(*s*) = α*sL*. In this coordinate, the whisker physical tip is located at *s*_tip_ = 1 − *L*_whisker_/*L*. Note that, owing to the truncation of the whiskers, *L* can be significantly larger than the actual whisker length *L*_whisker_.

The whisker profile is described in curvilinear angular coordinates as θ = θ(*s*) (Figure 2B). The whisker is embedded in the pad tissue down to 4–5 mm (Ebara et al., 2002), which strongly constrains the angular position of the whisker base. Although the pad does exhibit some level of elastic compliance, as established by Jenks et al. (2010) in awake rats (Jenks et al., 2010), here we assume the anchorage to be strictly rigid and thus impose θ(1) = θ_*b*_. The whisking process is modeled by imposing a rotation of the whisker around its base at constant rate γ. The base angle θ_*b*_ = θ(*s* = 1) thus grows linearly with time such that θ_*b*_(*t*) = γ*t*. The whisker is considered linearly elastic with a uniform Young's modulus *E* and density ρ. Any intrinsic (spontaneous) curvature and out-of-plane deformations are also ignored. When in contact with the object, the whisker is submitted to a frictional force *F* which is assumed to apply at a single point located at *s* = ε along the whisker. The present analysis is restricted to configurations where ε > *s*_tip_, which constrains the whisker to be locally tangent to the object surface. The orientation of the force *F* with respect to the direction normal to the whisker at *s* = ε is set by the friction angle ϕ = tan(μ) where μ is the friction coefficient (Persson, 2000). Within these hypothesis, the equation governing the whisker bending moment equilibrium reads (see “Methods” for the detailed derivation):

(1) $${\left(s^{4}\theta^{\prime }\right)}^{\prime }-\tilde{F}\cos(\phi -\theta +\theta (\varepsilon ))=0$$

where $\tilde{F}=4F/\left(\pi \alpha^{4}EL^{2}\right)$ is an adimensional force. The single contact point hypothesis further imposes that the moment is null at the contact point such that θ′(ε) = 0. For given values of θ_*b*_ (imposed by the rotation of the whisker base), the friction angle ϕ and the contact point ε, one can numerically compute a series of equilibrium whisker profiles by imposing different values of the contact angle θ(ε) < θ_*b*_. This method is first used to derive the quasi-static evolution of the whisker as it rolls over the edge of the rectangular obstacle. In this regime, the position of the contact point on the object is fixed. This condition yields, for each whisking angle θ_*b*_, a unique solution associated with a contact location ε along the whisker. As θ_*b*_ increases, the contact angle θ(ε) decreases and eventually vanishes. This time marks the onset of a second phase during which the whisker slides along the surface of the object. In this second regime, the contact angle θ(ε) is null while the radial distance *D* (the distance from the whisker base to the free surface) remains constant. Again, a unique solution, associated with a contact location ε, is obtained for each whisking angle θ_*b*_. When the contact point reaches the edge of the object, the whisker snaps off and then continues to rotate in air at a constant rotation rate.

**Figure 3:**
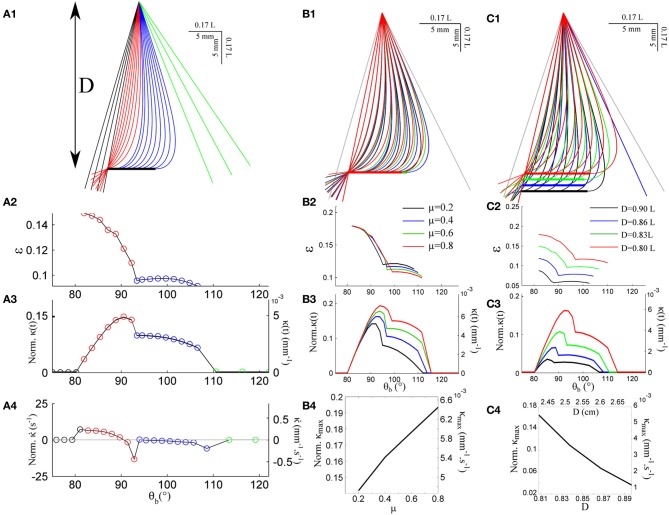
**Quasi-static evolution of a whisker rotating across a rectangular object. (A1)** Quasi-static sequence of whisker deformation for a friction coefficient μ = 0.4 and a radial distance *D* = 0.83 L. Different colors correspond to distinct phases: the whisker rotates in air (black), rolls over the obstacle edge (red), slides over the flat surface (blue) and, after detachment, rotates in air (green). The color code is conserved throughout the graphs. **(A2)** Evolution of the contact point location ε as a function of the base angle θ_*b*_ (θ_*b*_ step = 1.7°). **(A3)** Evolution of the whisker base moment κ(*t*) and **(A4)** its time derivative $\dot{\kappa }(t)$. **(B1–B3)** Same data shown for four different values of the friction coefficient μ. **(B4)** Maximum base moment as a function of μ. **(C1–C3)** Same data for different values of the radial distance *D*. **(C4)** Maximum base moment as a function of *D*.

Figure 3A shows the quasi-static evolution of the whisker profile for a friction coefficient μ = 0.4, a radial distance *D* = 0.83 L, and a rotation rate γ = 400°/s. During this sequence, the contact point position along the whisker varies within a small range 0.08 < ε < 0.15 (Figure 3A2). The graphs A3 and A4 display the evolution of the base moment κ(*t*) = θ′(*s* = 1) and its time-derivative $\dot{\kappa }(t)$. In all graphs, and throughout the article, double scales are used in order to show the data both in reduced (all lengths being normalized by *L*) and physical units. For the latter, a typical whisker length *L* = 3 cm is used. Figure 3B displays similar traces for various friction coefficients μ = {0.2, 0.4, 0.6, 0.8}. This range should encompass most physical situations (Persson, 2000). Although the associated profiles appear quite similar, increasing the friction coefficient yields a significant amplification of the base moment signal κ(*t*) as shown in Figure 3B4. One may notice that the effect becomes significant when the whisker is sufficiently deformed while the different graphs collapse in the early moments following the initial contact, i.e., when the whisker is essentially straight. The friction coefficient also controls the time (or base angle) at which the whisker detaches from the object. Although these frictional effects are significant, the base moment κ(*t*) appears to be mostly controlled by the radial distance *D* as shown in Figure 3C. Reducing this distance by 10% yields a five-fold increase of the maximum base moment experienced during the exploratory sequence. It thus seems unlikely that this quantity provides significant cue for the discrimination of surfaces exhibiting different frictional properties.

### Whisker resonant dynamics

The preceding section addressed the quasi-static evolution of the whisker as it is swept across a rectangular object. This sequence is expected to be valid for a massless whisker or at infinitely slow scanning speed (Quist and Hartmann, 2012). For a real whisker, however, inertia effects will induce significant deviations. In particular, the contact and detachment processes, which mark the transition between distinct mechanical conditions at the whisker tip, will trigger brief episodes of oscillations.

**Figure 4:**
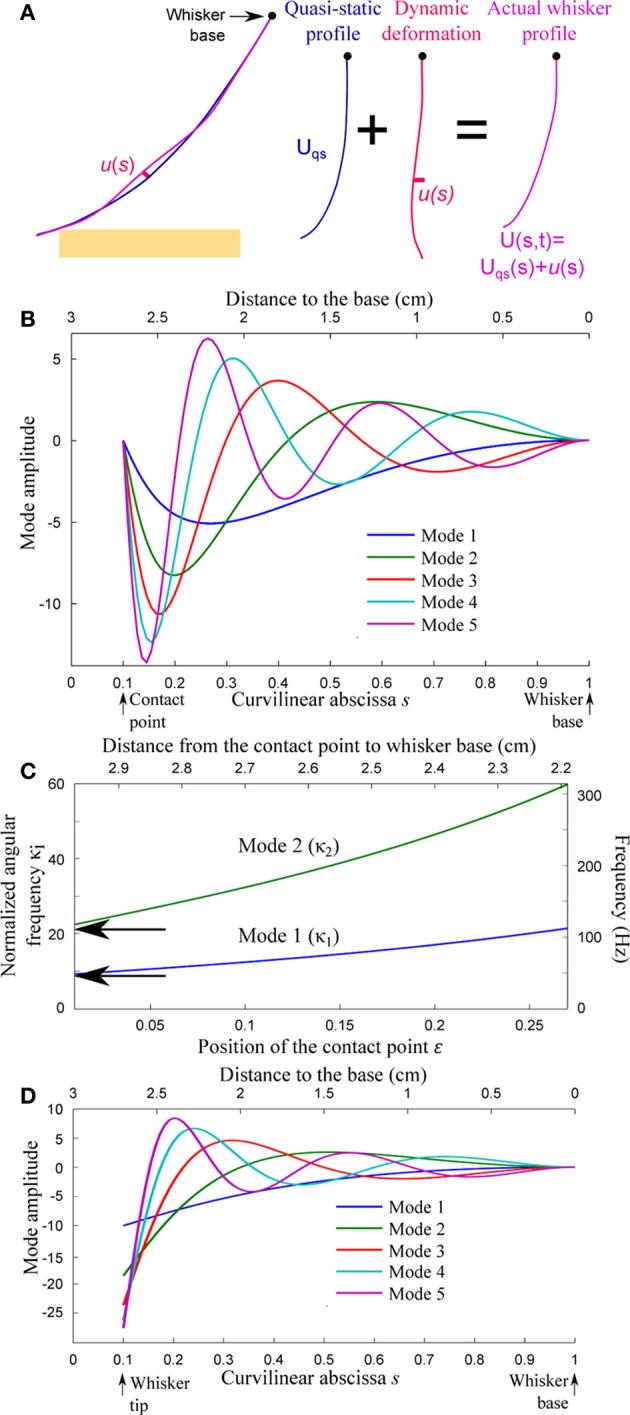
**Resonant properties of the whisker. (A)** The whisker deformation is decomposed into a quasi-static profile *U*_qs_(*s*, *t*) and a small amplitude deformation *u*(*s*, *t*) normal to the quasi-static profile. **(B)** First spatial modes for a whisker in contact at *s* = ε = 0.1 with boundary conditions *V*(ε) = *V*″(ε) = 0. **(C)** Resonant reduced angular frequency and resonant frequency (double scale) for the first two modes as a function of the contact location ε. **(D)** First spatial modes for a freely oscillating whisker (*V*″(ε) = (*s*^4^*V*′)″(ε) = 0).

These dynamic modulations are treated perturbatively in the form of a small displacement *u*(*s*, *t*) normal to the quasi-static profile sequence (see Figure 4A). We assume that both the quasi-static deformation and dynamic oscillations amplitude remain sufficiently moderate such that a small angle approximation (θ(*s*) « 1) can be implemented. The validity of this hypothesis, for both mechanical events, will be discussed *a posteriori*. In this limit, the classical Euler–Bernoulli equation that governs the force equilibrium normal to the whisker can be expanded around the quasi-static profile, yielding for *u*(*s*, *t*) (see Weaver et al., 1990):

(2) $$\frac{\partial^{2}}{\partial s^{2}}\left(EI\frac{\partial^{2}u}{\partial s^{2}}\right)+\rho A\frac{\partial^{2}u}{\partial t^{2}}=0$$

where *A* = π*r*^2^ is the whisker section area. This equation is re-written in reduced coordinates (all distances being expressed in unit of the ideal non-truncated whisker length *L*) in the form:

(3) $$\frac{\partial^{2}}{\partial s^{2}}\left(s^{4}\frac{\partial^{2}u}{\partial s^{2}}\right)+k^{2}s^{2}\frac{\partial^{2}u}{\partial t^{2}}=0$$

where $k=2\sqrt{\rho /E}L/\alpha$ is a time-scale characterizing the mechanical resonance of the isolated whisker: the fundamental resonance frequency of the freely vibrating whisker reads *f*_FRF_ = 1.39/k. In this expression, all lengths are expressed in units of *L*. Equation (3) is classically solved by separation of time and space variables: *u*(*s*, *t*) = *V*(*s*)*q*(*t*). The spatial term *V*(*s*) obeys the following equation:

(4) $${\left(s^{4}V^{\prime \prime }\right)}^{\prime \prime }-k^{2}\omega^{2}s^{2}V=0$$

The choice of boundary conditions (discussed below) sets the series of admissible angular frequencies ω_*i*_(*i* = 1, 2, …) = κ_*i*_/*k* and corresponding resonant spatial modes *V*_*i*_(*s*)(*i* = 1, 2, …). Each mode is associated with a harmonic equation of motion that reads:

(5) $$\ddot{q}+\omega_{i}^{2}q=0$$

In the absence of any knowledge on the underlying mechanism, dissipative processes are accounted for by introducing a linear damping term in the time-dependent Equation (5) with a mode-independent damping ratio ζ. The dynamic equation is thus rewritten as:

(6) $$\ddot{q}+2\zeta \omega_{i}\dot{q}+\omega_{i}^{2}q=0$$

Within this hypothesis, the general solution for the freely oscillating whisker finally reads:

(7) $$u(s,t)\text{​}=\text{​}\sum_{i}V_{i}(s)\left(\text{​}\alpha_{i}\cos(\sqrt{1\text{​}-\text{​}\zeta^{2}}\omega_{i}t)+\beta_{i}\sin(\sqrt{1\text{​}-\text{​}\zeta^{2}}\omega_{i}t)\text{​}\right)e^{-\zeta \omega_{i}t}$$

### Dependence of the whisker resonance frequency on the contact point Location

The resonant spatial modes *V*_*i*_(*s*) and associated angular frequencies ω_*i*_ depend on the boundary conditions. As already mentioned, the whisker is assumed to be rigidly anchored at its base, which imposes *V*(1) = *V*′(1) = 0. The different phases of the exploration correspond to distinct boundary conditions at the whisker tip.

- As the whisker rolls over the object's edge, the maintained contact imposes a constant position of the whisker at *s* = ε. Ignoring the inertia of the whisker tip (the region *s*_tip_ < *s* < ε), the moment at contact is null. The boundary conditions thus read *V*(ε) = *V*″(ε) = 0.
- As the whisker slides onto the object's flat surface, the maintained frictional contact imposes both a constant position and orientation of the whisker at *s* = ε, so that *V*(ε) = *V*′(ε) = 0.
- After detachment of the whisker from the object, the whisker oscillates freely in air. This yields a null moment and null force condition at the whisker tip, such that *V*″(ε) = (*s*^4^*V*″)′(ε) = 0.

For each value of the contact location ε in the range 0.01 < ε < 0.2, the first five modes *V*_1−5_(*s*) and associated adimensional angular frequencies κ_1−5_ are numerically computed using Mathematica v8.0 (Wolfram Research). Figures 4B,D display the spatial resonant modes obtained for ε = 0.1 and boundary conditions (a) and (c), respectively. Their strong asymmetry results from the tapered geometry of the whiskers. The resonant normalized angular frequencies κ_1_ and κ_2_ are shown in Figure 4C as a function of ε for boundary conditions (a). Both angular frequencies are found to increase as the contact point moves toward the base. The arrows indicate the corresponding angular frequencies for an isolated (freely vibrating) whisker. In order to express the resonant frequencies *f*_*i*_ = κ_*i*_/(2π*k*) in physical units (right axis scale), a typical time-scale *k* is computed using data from the literature (Hartmann et al., 2003). This value *k* = 30.4 ms is conserved throughout the article.

The slender geometry of the whisker confers it the property of a resonant oscillator, which thus acts as a mechanical band-pass filter. Neimark et al. observed resonance when shaking the whisker near its tip with a piezoelectric actuator (fixed–fixed boundary conditions) (Neimark et al., 2003). Although the boundary conditions were different between those data (fixed–fixed) and our model (fixed-pinned), the frequencies they reported for β and C1 fell within our predicted range (Figure 4C). Owing to their various lengths, the resonant frequencies of the freely vibrating whiskers span a wide range across the pad. This observation led Neimark et al. to propose a tonotopic scheme for texture encoding in which each whisker would transduce one particular spatial wavelength (Neimark et al., 2003). The present model may in part explain why this encoding scheme hypothesis failed to receive experimental validation so far. The effective resonant frequencies varies with the location of the contact point along the whisker and the way that the whisker is pinned and/or fixed at the contact point (see Figure 4C). The optimal transduction frequencies in real sensing situations, rather than being whisker specific, are thus expected to vary by a factor of up to 3 over the course of a single sensing task. It is tempting to suggest in reverse that the instantaneous resonant frequency may used by the rat to extract information about the distance from the pad to the touched object. However, the dependence being relatively weak (owing to the tapered geometry of the whisker), the spectral characteristic of the whisker dynamic is unlikely to play a significant role in the precise determination of radial distance.

### Shock against the object's edge

The resonant modes are now used to investigate the dynamics induced by the shock of the whisker against the object's edge. The whisker initially rotates in air at constant angular velocity γ around the whisker base. At time *t* = 0, the whisker makes contact with the object's edge at a position *s* = ε along the whisker. In line with previous observations (Hartmann et al., 2003), the collision is assumed to be inelastic such that the whisker tip remains in constant contact with the object at *t* > 0 (no rebound). The duration of the shock-induced oscillation is expected to damp out over a time period of order τ/ζ, where τ is the period of the fundamental mode. During this time, the contact point location ε, deduced from the quasi-static sequence, varies by less than 0.02 (0.002 after one resonant period). We ignore this minute change and assume ε to be constant, which allows one to describe the oscillating dynamics on a well-defined series of resonant modes.

The whisker profile *u*(*s*, *t*) at time *t* > 0 is decomposed as *U*(*s*, *t*) = *U*_qs_(*s*, *t*) + *u*(*s*, *t*) where *U*_qs_(*s*, *t*) is the quasi-static profile evolution and *u*(*s*, *t*) characterizes the shock-induced dynamics. By introducing this decomposition in Equation (3), *u*(*s*, *t*) is found to obey the dynamic equation (see “Methods”):

(8) $$\frac{\partial }{\partial s^{2}}\left(s^{4}\frac{\partial^{2}u}{\partial s^{2}}\right)+k^{2}s^{2}\frac{\partial^{2}u}{\partial t^{2}}=-k^{2}s^{2}{\ddot{U}}_{\text{qs}}(s,t)$$

One thus needs to compute the second time-derivative of the quasi-static profile ${\ddot{U}}_{\text{qs}}(s,t)$. Prior to the shock, the whisker experiences a solid rotation at constant rotation rate γ. Immediately after the shock, within the small deflection approximation, the quasi-static profile also evolves linearly in time (see “Methods”). The expression of ${\ddot{U}}_{\text{qs}}(s,t)$ can thus be written as:

(9) $${\ddot{U}}_{\text{qs}}(s,t)=\delta (t)\gamma \bar{U}(s)$$

where δ(*t*) is the Dirac function and $\bar{U}(s)$ is a normalized profile that depends on ε and γ (see “Methods” for analytical derivation). The dynamic term *u*(*s*, *t*) is decomposed onto the resonant modes *V*_*i*_(*s*) corresponding to boundary conditions *V*(ε) = *V*″(ε) = 0, in the form *u*(*s*, *t*) = ∑_*i*_*q*_*i*_(*t*)*V*_*i*_(*s*). Projecting expression (Equation 8) onto each mode *V*_*i*_(*s*), and using the orthogonality of the resonant modes, the mode amplitudes *q*_*i*_(*t*) are found to obey the dynamic equation [see Equations (36–41) in “Methods”]:

(10) $$\begin{array}{l} {\ddot{q}}_{i}+2\zeta \omega_{i}{\dot{q}}_{i}+\omega_{i}^{2}q_{i}=-\int_{\varepsilon }^{1}s^{2}{\ddot{U}}_{\text{qs}}(s,t)V_{i}(s)ds\\ \;=-\delta (t)\gamma \int_{\varepsilon }^{1}s^{2}\bar{U}(s)V_{i}(s)ds \end{array}$$

Notice that, as indicated before, a damping term is added to account for dissipative processes. After integration, the complete shock-induced dynamics reads:

(11) $$U(s,t>0)=\gamma t\bar{U}(s)-\gamma \sum_{i}V_{i}(s)\left(\int_{\varepsilon }^{1}s^{2}\bar{U}(s)V_{i}(s)ds\right)\times \frac{e^{-\zeta \omega t}\sin\left(\sqrt{1-\zeta^{2}}\omega_{i}t\right)}{\sqrt{1-\zeta^{2}}\omega_{i}}$$

**Figure 5:**
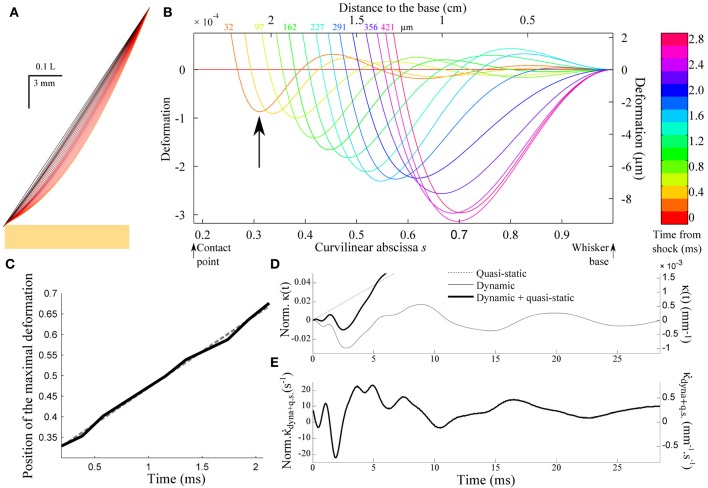
**Shock-induced whisker dynamics. (A)** Successive whisker profiles (from dark to light red) plotted at regular time interval (δ*t* = 1.06 ms) following the first whisker/object contact at ε = 0.2. The shock-induced oscillations are visible through the varying density of the profiles. **(B)** Whisker profiles in the reference frame of the whisker base (δ*t* = 0.2 ms). The distance indicated on each graph corresponds to the displacement of the contact point in μm. **(C)** Position of the maximal deformation as a function of the time elapsed since the shock (see arrow in **B**). The dotted line is the best linear fit and corresponds to a velocity 2.54*c*_wave_. **(D)** Time-evolution of the quasi-static κ_qs_(*t*) (dotted line) and dynamic κ_dyn_(*t*) (solid line) base moment. **(E)** Evolution of the time-derivative of the base moment $\dot{\kappa }(t)$.

Figure 5A shows the successive whisker profiles for a contact located at ε = 0.2. The damping factor is set at ζ = 0.1, consistent with values reported in the literature for the fundamental mode (Hartmann et al., 2003; Neimark et al., 2003). Figure 5B displays the same sequence in the reference frame of the whisker base (the imposed rotation of the whisker base has been subtracted). This graph illustrates how the shock at the tip of the whisker triggers a wave of deflection that travels up to the whisker base. Notice that the first maximum (indicated by an arrow in Figure 5B) is negative, i.e., opposite to the long time scale whisker deflection induced by the object. The position of the first minimum displays a linear dependence with the time elapsed since the shock (Figure 5C). This constant wave velocity results from the tapered geometry, since a $\sqrt{t}$ dependence is expected in the case of a cylindrical rod (Audoly and Neukirch, 2005). From dimensional analysis of Equation (3), the wave velocity is expected to be of order *c*_wave_ = α*c* where α is the cone angle and $c=\sqrt{E/\rho }$ is the sound velocity in the bulk material. The apparent wave velocity obtained by linear fitting on graph C is consistently found to be 2.54*c*_wave_ (5.02 m/s in physical units). Figure 5D shows the evolution of the moment κ(*t*) = ∂^2^*U*/∂*s*^2^(*s* = 1,*t*) at the base of the whisker (black solid line). The graph also displays both the dynamic (κ_dyn_(*t*)= ∂^2^*u*/∂*s*^2^(*s* = 1, *t*), gray solid line) and quasi-static (κ_qs_(*t*) = ∂*U*^2^_qs_/∂*s*^2^(*s* = 1, *t*), dotted line) components for comparison. Although the maximum amplitude of the deflection wave is of the order of 15 μ m, i.e., a small fraction of the whisker base diameter, it yields a significant negative dip in the whisker base curvature dynamics. The time-derivative $\dot{\kappa }(t)$ signal (Figure 5E) in turn exhibits a clear signature of the whisker/object contact in the form of large amplitude oscillation with a peaked maximum occurring at a time τ_peak_ ≈ 4 ms after the shock.

**Figure 6:**
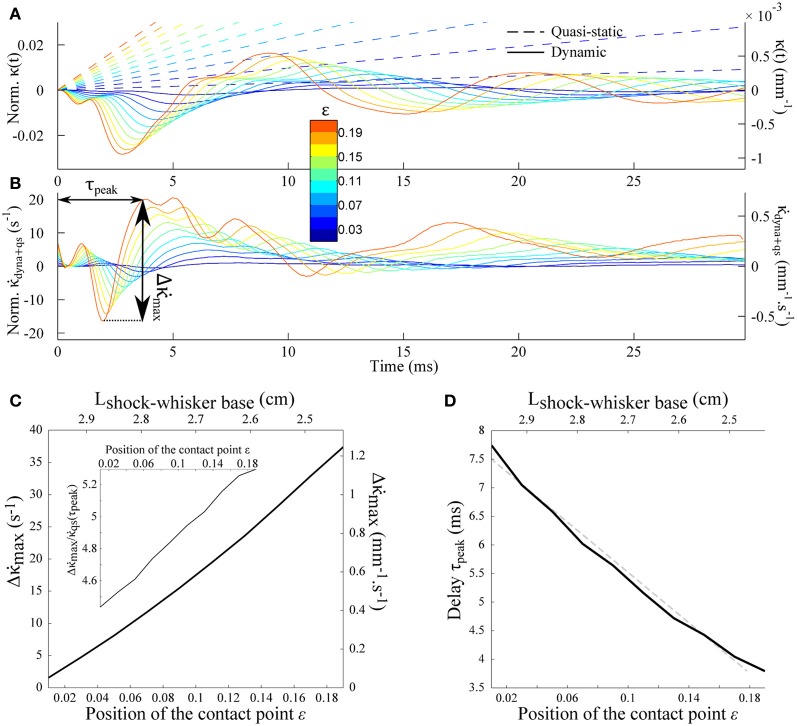
**Shock-induced mechanical signal at the whisker base. (A)** Time-evolution of the quasi-static κ_qs_(*t*) (dotted line) and dynamic κ_dyn_(*t*) (solid line) base moment for different contact location (color code). **(B)** Evolution of the time-derivative of the base moment $\dot{\kappa }(t)$. **(C)** Peak amplitude $\Delta {\dot{\kappa }}_{\text{max}}$ as a function of the contact location. The inset shows the same data normalized by the quasi-static component ${\dot{\kappa }}_{\text{qs}}(\tau_{\text{peak}})$. **(D)** Delay τ_peak_ (see arrows on panel **B**) as a function of the contact location. The dotted line corresponds to the best linear fit, yielding an effective velocity 0.69*c*_wave_.

Equation (11) indicates that the peak amplitude $\Delta {\dot{\kappa }}_{\text{max}}$ should be linearly proportional to the rotation rate γ. It also increases as the contact point moves toward the whisker base i.e., for shorter radial distances (Figures 6A–C). Notice that the peak amplitude remains larger than the quasi-static value ${\dot{\kappa }}_{\text{qs}}(\tau_{\text{peak}})$ (Inset). The last graph of Figure 6 shows the delay τ_peak_ between the shock event and the arrival of the mechanical signal at the whisker base as a function of the contact location. The dependence is quasi-linear within the range of ε explored, which allows us to extract an approximate velocity 0.69*c*_wave_ (1.36 m/s in physical units) of the same order as the wave velocity determined in Figure 5.

In the range of parameters explored, the maximum angular deflection of the whisker during the process is 0.06. This value validates the small angle hypothesis underlying the present analysis (Birdwell et al., 2007).

### Experimental measurements of shock-induced oscillation

**Figure 7:**
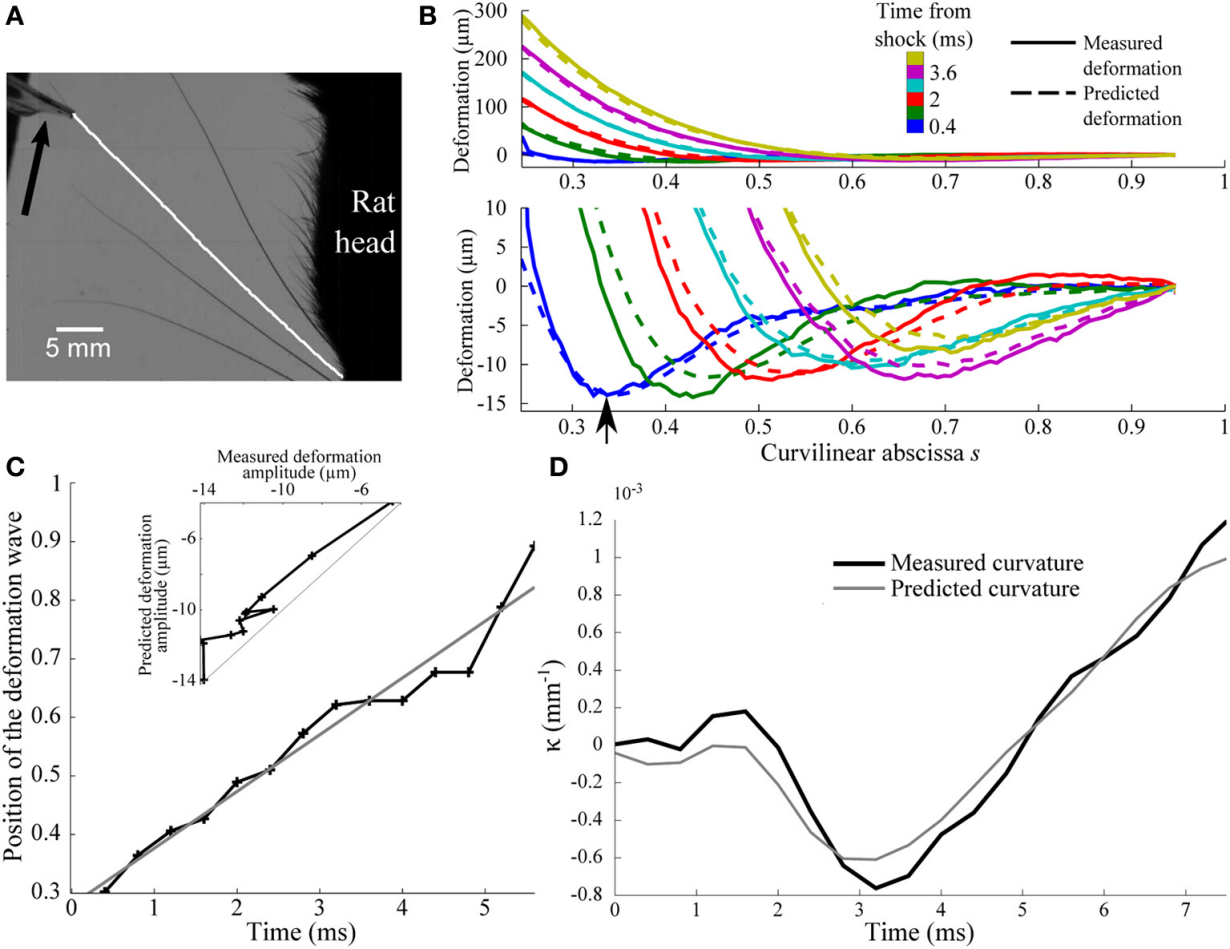
**Experimental shock-induced whisker dynamics. (A)** Snapshot of the whisker/object initial contact (sampling rate 2.5 kHz) between the vertical bar and the β whisker. The result of the whisker tracking is superimposed (white line) on the frame. The direction of movement of the bar is indicated by an arrow. **(B)** Whisker profiles in the reference frame of the whisker base (δ*t* = 0.8 ms). The bottom panel is a magnified view of the low amplitude deformation in the top panel. Solid and dotted lines correspond to experimental and theoretical profiles, respectively. **(C)** Position of the maximal deformation as a function of the time elapsed since the shock (see arrow in **B**). The gray line is the best linear fit and corresponds to a wave velocity 5.6 m/s. **(D)** Time-evolution of the experimental (solid line) and predicted (gray line) κ(*t*) base curvature signal.

These predictions are tested experimentally on an anesthetized rat using high-speed videography (see “Methods”). The rat, and thus the whisker base, are maintained fixed while a thin bar is moved at constant speed *V*_bar_ = 60 mm/s against the whisker tip (Figure 7A). We used a constant speed and not a tap since in that case a sudden acceleration would happen, which is a different condition and ethologically more unlike to happen. The bar is vertical and fixed by its edge to a rectilinear motor. During the early instants following the shock, which are analyzed here, the whisker did not slip onto the bar such that the contact point along the whisker can be considered invariant. Whisker movements are captured using a bird's-eye view high-speed camera operating at 2.5 kHz (Figure 7A). Whisker centerline profiles are tracked within each frame between the fur and the bar with a custom-designed semi-automatic script. The shock-induced whisker deflection profile *u*(*s*, *t*) is obtained by subtracting, for each frame, the whisker intrinsic profile as determined from images recorded before the shock.

The resulting sequence of shock-induced whisker deflections is shown in Figure 7B for a β whisker (length 46 mm) during the first 3 ms from the instant of collision. Consistent with the model, a deflection wave is observed characterized by a negative maximum deflection propagating upward. As expected, the wave appears to propagate at constant speed as indicated by the linear dependence of the location of the maximum deflection with time (Figure 7C). This allows us to extract an effective wave velocity equal to 5.6 m/s, in close agreement with the typical value predicted before.

In order to more quantitatively compare these results with the biomechanical model, we decomposed the whisker dynamics into rapid resonant oscillations superimposed onto a slow quasi-static sequence of deformation imposed by the moving bar. The quasi-static evolution *U*_qs_(*s*, *t*) is first evaluated using the profiles measured at long time scale, i.e., when the relative contribution of the dynamic oscillation is expected to be negligible (*t* > 8 ms). As expected, in this regime, *U*_qs_(*s*, *t*) can be linearized as $U_{\text{qs}}(s,t)=V_{\text{bar}}t\bar{U}(s)$. The normalized profile $\bar{U}(s)$ is well fitted by the static theoretical profile in the limit of small deflection (see “Methods”). The best fit yields a value of ε = 0.25. Using the long time-scale normalized profile $\bar{U}(s)$, the complete sequence of whisker deflection *u*(*s*, *t*) is computed using the same scheme as described earlier [adapted to the linear displacement configuration (see “Methods”)]. The comparison, for each parameter, is shown in Figures 7B–D. The model quantitatively captures not only the wave propagation dynamics (*r*^2^ = 0.96, *p* < 2.10^−9^) (Figure 7C), but also the amplitude of the maximum deflection (*r*^2^ = 0.94, *p* < 2.10^−8^) (inset in Figure 7C) and, most importantly, the base curvature signal (*r*^2^ = 0.94, *p* < 2.10^−8^) (Figure 7D).

Identical measurements were performed for a C1 whisker (length 36 mm; ε = 0.15). The wave dynamics was found consistent with the model prediction (*r*^2^ = 0.95, *p* < 10^−5^) with a wave velocity of 5.8 m/s, close to the value obtained for the β whisker. The measured wave amplitude appeared significantly lower than predicted during the first ms following the shock, resulting in low values of correlation for this parameter (*r*^2^ = 0.41, *p* = 0.06). After this initial period, however, a very consistent match was recovered (*r*^2^ = 0.97, *p* < 5.10^−5^). The measured curvature signal at the whisker base being rather unaffected by the early deflections of the whisker tip, it was found to agree with the prediction for all the duration of the process (*r*^2^ = 0.65, *p* = 0.0027).

### Consequence for event-based object position encoding

As the rat repetitively whisks onto an object, it produces a series of shocks. The present work demonstrates that each of them triggers, a few ms after contact, a characteristic signature in the base curvature signal which can be quantitatively predicted using a first order mechanical model of the whisker. The resulting mechanical stimulation at the whisker pad should be sufficiently intense to trigger a clear neural response. As displayed in Figure 7, the whisker peak base curvature is of the order of 10^−3^ mm^−1^ and its maximum time-derivative varies up to 1 mm^−1^. s^−1^. Stimuli of comparable intensity have been shown to elicit reliable cortical discharges (O'Connor et al., 2010b; Huber et al., 2012).

Notice that the peak in the base curvature time derivative has an opposite sign and a larger amplitude compared to the long-time component, which may explain how so-called touch cells may specifically respond to first contact (Szwed et al., 2003). It has been recently proposed that these cells may mediate the coding of object angular position with respect to the pad through the precise timing of the shock event within the whisking cycle (Knutsen and Ahissar, 2009). The present biomechanical analysis allows us to estimate how the pre-neural whisker transduction contributes to the horizontal resolution of such an encoding scheme.

If one assumes that the neural response is triggered by the maximum of $\dot{\kappa }(t)$, the jitter Δ*T* in the mechanoreceptor response (the dispersion in spike timing) should be a fraction of the fundamental resonant period, i.e., a few ms. With a characteristic rotation rate during whisking of order γ = 400°, this yields an angular resolution of γΔ*T* ≈ 1° consistent with available behavioral data (Knutsen et al., 2006). This crude evaluation implicitly ignores the lag between the shock and the arrival of the deflection wave at the whisker base that elicits the mechanoreceptors' response. This delay was shown to be of the order of *c*_wave_*L*_contact−base_ where *L*_contact−base_ is the arc length between the contact point and the whisker base. This lag effect induces an additional error of up to a few degrees on the angular position of the object if its radial distance is unknown.

One may therefore suggest that the radial distance is evaluated using a parallel coding channel. One possible scenario, as originally proposed by Szwed et al. (2003), relies on the intensity of the mechanical signal which appears to decay rapidly with the radial distance, as shown in Figure 6C. By combining both information (timing and intensity of the shock-induced whisker base mechanical signal), a precise localization of the object can be recovered (Knutsen and Ahissar, 2009).

### Detachment

We now turn to the detachment process that occurs when the whisker tip reaches the second edge of the object. We note *t* = 0 the time at which the whisker snaps off. At *t* > 0, the whisker detaches from the object then oscillates freely in air. The profile is decomposed as *U*(*s*, *t*) = *U*_qs_(*s*, *t*) + *u*(*s*, *t*) where *U*_qs_(*s*, *t*) is the quasi-static evolution. The continuity of the position and velocity profiles at time *t* = 0 imposes:

(12) $$u(s,t=0)=\Delta U_{\text{qs}}(s)$$

(13) $$\dot{u}(s,t=0)=\Delta {\dot{U}}_{\text{qs}}(s)$$

where Δ*U*_qs_(*s*) = *U*_qs_(*s*, 0^−^) − *U*_qs_(*s*, 0^+^) and $\Delta {\dot{U}}_{\text{qs}}(s)={\dot{U}}_{\text{qs}}(s,0^{-})-{\dot{U}}_{\text{qs}}(s,0^{+})$. The term *u*(*s*, *t*) is decomposed along the resonant modes with boundary conditions *V*″(ε) = (*s*^4^*V*″)′(ε) = 0 as in Equation (7):

(14) $$u(s,t)=\sum_{i}V_{i}(s)(\alpha_{i}\cos(\sqrt{1\text{​}-\text{​}\zeta^{2}}\omega_{i}t)+\beta_{i}\sin(\sqrt{1\text{​}-\text{​}\zeta^{2}}\omega_{i}t))e^{-\zeta \omega_{i}t}$$

Projecting Equations (12) and (13) on each spatial mode, the coefficients α_*i*_ and β_*i*_ can be written, in the limit ζ « 1:

(15) $$\alpha_{i}=\int_{\varepsilon }^{1}s^{2}\Delta U_{\text{qs}}(s)V_{i}(s)ds$$

(16) $$\beta_{i}=\frac{1}{\omega_{i}}\int_{\varepsilon }^{1}s^{2}\Delta {\dot{U}}_{\text{qs}}(s)V_{i}(s)ds$$

In order to obtain an expression of Δ*U*_qs_ and $\Delta {\dot{U}}_{\text{qs}}$, the quasi-static profiles are computed at time {−δ*t*, 0^−^, 0^+^, δ*t*} with δ*t* << 1/γ. This allows us to obtain linearized expressions of the quasi-static profile sequences around *t* = 0, before and after the shock, in the form:

(17) $$U_{\text{qs}}(s,t<0)=U_{\text{qs}}(s,0^{-})+\frac{U_{\text{qs}}(s,0^{-})-U_{\text{qs}}(s,-\delta t)}{\delta t}t$$

(18) $$U_{\text{qs}}(s,t>0)=U_{\text{qs}}(s,0^{+})+\frac{U_{\text{qs}}(s,\delta t)-U_{\text{qs}}(s,0^{+})}{\delta t}t$$

This yields:

(19) $$\Delta U_{\text{qs}}(s)=U_{\text{qs}}(s,0^{-})-U_{\text{qs}}(s,0^{+})$$

(20) $$\Delta {\dot{U}}_{\text{qs}}(s)=\frac{1}{\delta t}(U_{\text{qs}}(s,0^{-})-U_{\text{qs}}(s,-\delta t)-U_{\text{qs}}(s,\delta t)+U_{\text{qs}}(s,0^{+}))$$

**Figure 8:**
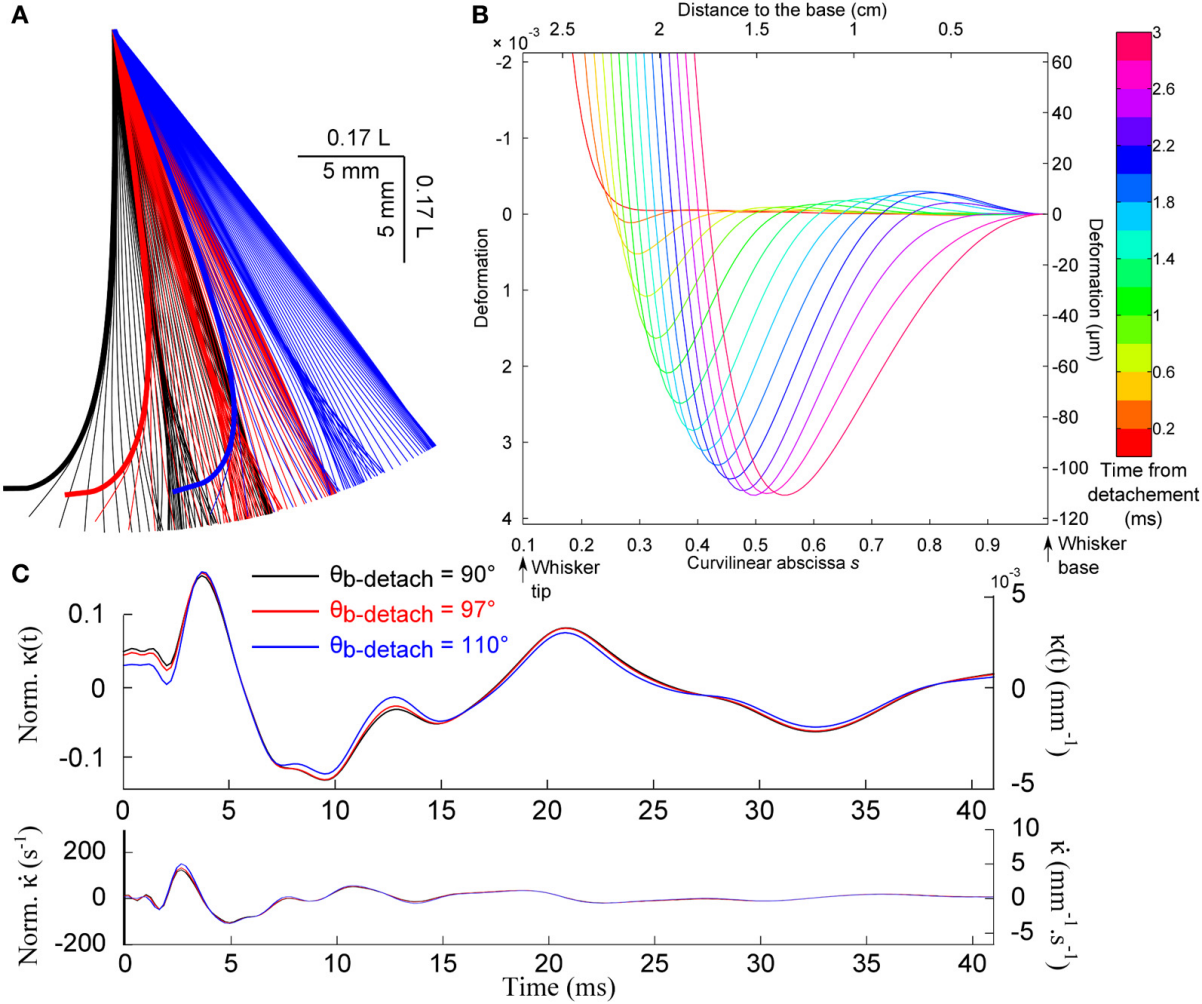
**Detachment-induced whisker dynamics. (A)** Consecutive whisker profiles (δ*t* = 0.6 ms) for different base angle θ_*b*_ at detachment. **(B)** Dynamic profiles [the quasi-static evolution has been subtracted (δ*t* = 0.2 ms)]. **(C)** Base moment κ(*t*) and its time-derivative signals for different base angle at detachment.

Figure 8A shows the evolution of the whisker profile just after the detachment, for different base angle θ_*b*_ at detachment. When the quasi-static evolution is subtracted (graph B), one recovers a wave propagation mechanism qualitatively similar to that observed after the shock (wave speed of order 3.5 m/s). This event produces a characteristic signature at the whisker base shown in Figures 8B,C. Notice that the mechanical signal is rather insensitive to the whisker base angle prior to the detachment.

As indicated earlier, the present analysis is based on the assumption that the whisker remains weakly deformed during the whole process. As shown in Figure 8A, this hypothesis becomes valid only a few hundreds of microseconds after the event is triggered. At very early time, and for large angular and small radial distances to the object, the tip of the whisker exhibits large deflection angle. However, owing to the conical shape of the whisker, the large deformation appears to be confined to the very end of the whisker: in the range of configurations explored, 80% of the whisker, i.e., 99% of the mass, displays a deflection angle smaller than 0.5. The small angle approximation should therefore provides a reasonable first order approximation of the detachment dynamics for all situations explored.

The whisker detachment from the object also triggers a deflection wave that propagates upward at a speed comparable to that observed for the shock event. The resulting signal at the whisker base appears to be ≈ 10 times more intense than for the shock. It should thus trigger significant neural response, as confirmed by the existence of so-called detachment cells whose response is specifically triggered by such events (Szwed et al., 2003). Our study shows that an increase in the friction coefficient delays the detachment process (Figure 3B). The precise timing of the detachment-induced signal may thus provide an indication on the surface frictional properties. Such a scenario would however, require the independent knowledge of the angular position of the object edge.

### Vibrissae can be assumed to be uniformly damped over all spatial modes

A key assumption in the development of the present model is that the damping coefficient of the vibrissa is constant over all spatial modes. Previous experimental studies, however, have shown that magnification ratio increases with mode number (Hartmann et al., 2003; Neimark et al., 2003). Specifically, the bottom plot of Figure 4 of Hartmann et al. (2003), and Figure 4 of Neimark et al. (2003) (e.g., the β whisker) both show an increase in peak magnitude with mode number. Note that Neimark et al. chose to plot the curve assuming constant amplitude (instead of constant acceleration), so each peak must be scaled by the frequency squared to demonstrate the increase with mode number. This experimental finding has until now been unexplained, although Hartmann et al. (2003) suggested that it was likely in part due “to nonlinear effects of viscous and/or hysteretic damping.”

Here we show that, at odds with the authors' proposed interpretation, a linearly damped model of whisker with a mode-independent damping ratio, as assumed in the present work, allows one to correctly capture this measurement. It also illustrates how the mode-decomposition approach introduced in the present study, may allow to quantitatively re-analyze earlier data on whisker dynamics.

**Figure 9:**
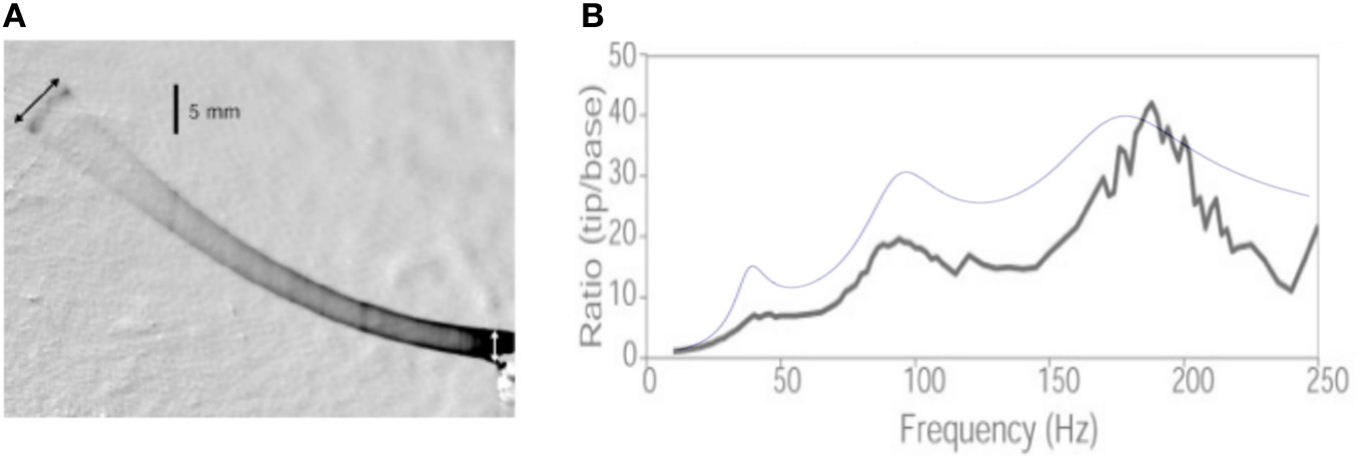
**Comparison with Hartmann et al. (2003). (A)** Figure 1 from Hartmann et al. (2003), showing the whisker vibration driven by the base imposed sinusoidal motion. **(B)** Resonance curves: the black line is the experimental measurements of the tip to base amplitude ratio reported by Hartmann et al. The blue line is the result of the present calculation.

Throughout the calculation, we use the same notation as in the present article. The text indicates that the whisker has a length *L*_whisker_ = 53 mm, a base radius *b* = 105 μm and a tip radius *r*_tip_ = 5.5 μm. Assuming a conical shape, this yields ϵ = *r*_tip_/*b* = 0.05. Using the Young modulus *E* = 3.02 GPa and ρ = 1.14 mg/mm^3^ indicated in the article, the values for the (undamped) resonant frequencies can be computed and read: *F*_i_ = {37.9, 93.4, 175.4 Hz}. The whisker base is forced with a sinusoidal motion [Figure 9A reproduced from Hartmann et al. (2003)], starting at *t* = 0:

(21) $$A_{\text{base}}(t)=A_{\text{base}}\sin(\omega t)$$

The whisker response is derived using the same scheme as detailed in the present article. The displacement sequence is broken into *U*(*s*, *t*) = *U*_qs_(*s*, *t*) + *u*(*s*, *t*) where the first term represents the massless whisker response: *U*_qs_(*s*, *t*) = *A*_base_ sin(ω*t*). The dynamic term can thus be written as:

(22) $$u(s,t)=\sum_{i}V_{i}(s)\int_{\epsilon }^{1}s^{2}V_{i}(s)ds\int_{0}^{t}G(t-t^{\prime }){\ddot{U}}_{\text{qs}}(s,t^{\prime })dt^{\prime }$$

(23) $$=-A_{\text{base}}\sum_{i}V_{i}(s)\int_{\epsilon }^{1}s^{2}V_{i}(s)ds\int_{0}^{t}G(t-t^{\prime })\omega^{2}\times \sin(\omega t^{\prime })dt\prime$$

where *G*(*t*) is the Green's function of the resonant system (Equation 49).

The time-dependent term in Equation (23) can be analytically integrated. It oscillates at the driving frequency with an absolute peak-to-peak amplitude that can be written as 2*F*(ω/ω_*i*_, ζ). Due to the resonant nature of the system, the spectrum is dominated by the closest resonant mode. We thus approximate the maximal amplitude of the response signal with the sum of the maximal amplitude of each mode signal. The ratio between the whisker tip oscillation amplitude (the peak-to-peak amplitude of signal *u*(*s*, *t*) measured at *s* = ε) and the driving base amplitude 2*A*_base_ thus reads:

(24) $$R(\omega )\approx 1+\sum_{i}\left[V_{i}(\epsilon )\int_{\epsilon }^{1}s^{2}V_{i}(s)ds\right]F(\omega /\omega_{i},\zeta )$$

The dependence of the maxima of the response signal with mode number thus depends primarily on the series of prefactors $V_{i}(\epsilon )\int_{\epsilon }^{1}s^{2}V_{i}(s)ds$, such that no conclusion can be drawn without prior knowledge of the resonant spatial modes. In the present case, this prefactor reads, for the first three modes: {3.7, 6.5, 7.6} and thus does increase with the mode number. Notice that the evolution of the peak amplitude results from the conical shape of the whisker—it would decrease for a cylindrical rod. Figure 9B compares the prediction obtained for a damping ratio of 0.15 (a characteristic value given by the authors) identical for all three modes. Although no adjustable parameter has been used to produce this plot, it does captures the evolution of the whisker tip response. The slight over-estimation of the two first modes can be due to the fact that the whisker displays significant spontaneous curvature, such that the small angle limit is not strictly valid.

It thus appears that the data reported in previous experimental studies (Hartmann et al., 2003; Neimark et al., 2003) are, to first order, consistent with a linear damping model with a unique damping ratio for all modes.

## Discussion

The biomechanical model presented here provides a generic method to describe the mechanical transduction operated by the whisker in realistic conditions of exploration. The theoretical approach consists of successively computing the slow (quasi-static) evolution of the whisker and the rapid resonant dynamics. It provides prediction of the complete whisker deflection, and in particular of the mechanical signal elicited in the follicle which conveys the relevant peripheral input for the mechanoreceptors. It is therefore directly amenable to experimental comparison as illustrated in the present article in the case of first contact. In this configuration, we were able to characterize the dynamic of the deflection wave with micron-scale resolution by subtracting the static configuration of the whisker prior to the event. For the detachment case, the deflection wave proved to be difficult to observe with such precision owing to the continuous evolution of the whisker quasi-static profile prior to the event.

Beyond the specific context in which it is implemented in the present study, this biomechanical model may constitute a useful tool for understanding the neural encoding of tactile information. It may in particular guide the type of stimuli that needs to be played at the whisker base during electrophysiological recordings in the somatosensory cortex. It should also find direct applications in robotic rats by easing the development of decoding algorithms allowing one to extract relevant physical information from the whisker inputs (Kim and Möller, 2007). This approach, unlike finite-element models, allows one to clearly identify the consequences of whiskers' resonant properties.

The model is based on several simplifying assumptions and should therefore be considered as a first order description of the whisker dynamics. First, the whisker spontaneous curvature and out-of-plane deflections are ignored. For moderately curved whisker, the spontaneous curvature can be accounted for when deriving the quasi-static sequence by modifying Equation (1) as explained in the “Methods” section. The prediction of the dynamic model itself does not depend on the resting state. However, for largely deformed whisker, the paradigmatic scenario envisioned here might be changed. In particular, the hypothesis of a shock normal to the whisker axis might be unrealistic for strongly concave-forward whiskers (Quist and Hartmann, 2012). For such vibrissae, the out-of-plane deflections are bound to be also significant.

Second, the whisker is assumed to exhibit a conical shape and to be linearly elastic, with uniform density and elastic modulus. It has been recently reported that the Young's modulus *E* may vary significantly along the whisker, owing to its internal structure (Quist et al., 2011), although conflicting results exist (Carl et al., 2012). However, one should notice that the modulus appears in the equation through the bending stiffness which scales as *r*^4^*E* (*r* whisker radius), so that the most critical hypothesis should actually lie in the conical shape assumption. Indeed, a 10% deviation to the conical shape is expected to induce a larger deviation than a 40% variation of *E* along the whisker.

Third, the model assumes linear damping, characterized by a unique parameter ζ in the dynamic equation. This *ad-hoc* introduction of a damping term is not based on any sort of physical modeling and reflects the absence of prior knowledge on the nature of dissipative processes. At least five different mechanisms may contribute to damping: visco-elastic dissipation in the whisker itself, mechanical radiation in the tissue, air friction, interfacial dissipation at the whisker/substrate contact, visco-elastic dissipation in the follicle. In the absence of well-established mechanical descriptions of the dissipative mechanism, it is common for lightly damped systems (i.e., effective damping ratio <0.2) to assume proportional damping (Weaver et al., 1990), as done in the present work. One should notice that typical damping ratios reported in the literature for the fundamental mode lies in the range 0.1 < ζ < 0.2 such that the resonant system is well within the underdamped limit. As a result, the resonant frequency is not strongly affected by the precise choice of the damping ratio. During the review process, one of the referees pointed to us that the linear damping hypothesis with a unique damping ratio appears at odds with resonance curves experimentally obtained by Hartmann et al. on isolated whiskers (Hartmann et al., 2003). When re-analyzed using the resonant modes description, these data are in fact consistent with this hypothesis as demonstrated in the “Methods” section.

Within this set of hypothesis, it appears that the whisker transduction mechanism depends on the whisker intrinsic properties through only three independent parameters: (1) the length of the equivalent non-truncated whisker *L* (2) the wave velocity $c_{\text{wave}}=\alpha \sqrt{E/\rho }$ that characterizes the speed at which information is conveyed from the whisker tip to the follicle, and (3) the damping factor ζ that reflects the dissipative processes (the intrinsic time-scale *k* is the product 2*c*_wave_*L*). Notice that the Young's modulus *E*, the density ρ, and the cone angle α vary in a small range across the whisker pad (Hartmann et al., 2003; Carl et al., 2012; Voges et al., 2012) so that *c*_wave_ should weakly depend on the whisker identity. Similarly, the values reported for the damping factor appear to lie within a rather small range (Hartmann et al., 2003; Neimark et al., 2003). When displayed in reduced coordinates (all lengths being expressed in units of *L*), the present results should therefore provide a reasonable description of the rapid whisker dynamics, regardless of the whisker identity.

The last and most limiting hypothesis of the proposed dynamic model is that its validity requires the whisker deflection to remain small. For large deflections, the dynamic equation should include additional terms that reflect the coupling between the quasi-static (deformed) state and the oscillating dynamic. This correction might become significant when one attempts to describe situations in which the whisker vibrates around strongly pre-deformed configurations, such as encountered during exploration of textured surfaces for instance. At the expense of significant mathematical developments, it should be possible to derive the resonant spatial modes in this regime and then implement the same theoretical scheme. However, as illustrated with the detachment process, the whisker tapered shape tends to confine the large deflection to the very end of the whisker even for large angular distances. One may thus hope that this first order description provides a good approximation in most realistic exploratory conditions. We postpone the discussion of this regime of whisker oscillations during sliding to a forthcoming publication.

## Methods

### Equilibrium profile of a frictional whisker

The whisker is modeled as a slender tapered rod of length *L* and maximum base radius *b*. The cone angle *b*/*L* is noted α. The whisker base is rigidly clamped with a fixed angle θ(*s* = 1) = θ_*b*_ and submitted to a frictional force *F* assumed to be applied at a single point located at *s* = ε along the whisker. The present analysis is restricted to configurations where ε > *s*_tip_ which constrains the whisker to be locally tangent to the object surface. The orientation of the friction force *F* with respect to the direction normal to the whisker at *s* = ε is set by the friction angle ϕ = tan(μ) where μ is the friction coefficient (see Figure 2B). Forces balance along the whisker yields an expression for the normal *N*(*s*) and tangential force *T*(*s*) at location *s* > ε:

(25) N(s)=Fcos(ϕ−θ+θ(ε))

(26) T(s)=Fsin(ϕ−θ+θ(ε))

The momentum equilibrium further imposes:

(27) $$\frac{\partial M}{\partial s}-N(s)=0$$

where the bending moment can be expressed as $M(s)=EI\theta^{\prime },I=\frac{\pi \alpha^{4}s^{4}}{4}$ is the area moment of inertia and *E* is the Young's modulus. From Equations (25) and (27), one obtains the dimensionless static equilibrium equation:

(28) $${\left(s^{4}\theta^{\prime }\right)}^{\prime }-\tilde{F}\cos\left(\phi -\theta +\theta (\varepsilon )\right)=0$$

where all distances are scaled by the length of the non-truncated cone *L* and the reduced force $\tilde{F}=4F/\left(\pi \alpha^{4}EL^{2}\right)$. Notice that the intrinsic in-plane whisker curvature θ_int_′(*s*) could be taken into account by substituting the first (derivative) term by (*s*^4^(θ′ − θ_int_′))′. In the present study, the intrinsic whisker curvature is supposed to be null. Equation (28) is solved numerically using Mathematica v8.0 (Wolfram Research) through integration of the following equation:

(29) $$\frac{\partial }{\partial s}\left(\frac{{\left(s^{4}\theta^{\prime }\right)}^{\prime }}{\cos\left(\phi -\theta +\theta (\varepsilon )\right)}\right)=0$$

We assume the whisker to be rigidly clamped in the pad such that θ(1) = θ_*b*_. The friction force is assumed to apply at a single location so that the bending momentum is null at the contact point: θ′(ε) = 0. For any values of the friction coefficient μ, maximum angle θ_*b*_, and contact point location ε, one finds a unique solution when further imposing the contact angle θ(ε) < θ_*b*_.

For weakly deformed whiskers, a small angle approximation of Equation (29) can be used which reads, in Cartesian coordinates:

(30) $${\left(s^{4}U^{\prime \prime }\right)}^{\prime \prime }=0$$

### Orthogonality of the resonant modes

Multiplying Equation (4) for mode *i* by *sV*_*j*_ and integrating between ε and 1 gives the equality:

(31) $$\int_{\varepsilon }^{1}{\left(s^{4}V_{i}^{\prime \prime }\right)}^{\prime \prime }V_{j}ds=k^{2}\omega_{i}\int_{\varepsilon }^{1}s^{2}V_{i}V_{j}ds$$

Integrating the left member by parts (twice) yields:

(32) $${\left[{\left(s^{4}V_{i}^{\prime \prime }\right)}^{\prime }V_{j}\right]}_{\varepsilon }^{1}-{\left[s^{4}V_{i}^{\prime \prime }V_{j}^{\prime }\right]}_{\varepsilon }^{1}+\int_{\varepsilon }^{1}s^{4}V_{i}^{\prime \prime }V_{j}^{\prime \prime }ds=k^{2}\omega_{i}\int_{\varepsilon }^{1}s^{2}V_{i}V_{j}ds$$

The boundary conditions involve that the bracket terms are null. One can rewrite the same expression by swapping *i* and *j*. If we now subtract both equations, we obtain:

(33) $$(\omega_{i}^{2}-\omega_{j}^{2})\int_{\varepsilon }^{1}s^{2}V_{i}V_{j}ds=0$$

The spatial mode *V*_*i*_(*s*) are normalized such that $\int_{\varepsilon }^{1}s^{2}V_{i}^{2}=1$. This yields the orthogonality property of the resonant modes:

(34) $$\begin{array}{l} \int_{\varepsilon }^{1}s^{2}V_{i}V_{j}ds=0\text{ for }i\neq j\\ \int_{\varepsilon }^{1}s^{2}V_{i}V_{j}ds=1\text{ for }i=j \end{array}$$

This relationship has practical consequences. First, let's consider a dynamic evolution of the whisker in the form: *u*(*s*, *t*) = ∑_*i*_*q*_*i*_(*t*)*V*_*i*_(*s*)*ds*. The amplitude of mode *i* can be directly computed as:

(35) $$q_{i}(t)=\int_{\varepsilon }^{1}s^{2}V_{i}(s)u(s,t)ds$$

Second, if we now consider a situation where the whisker oscillates through the application of a distributed normal force gradient *f*(*s*, *t*), Equation (3) reads:

(36) $$\frac{\partial }{\partial s^{2}}\left(s^{4}\frac{\partial^{2}u}{\partial s^{2}}\right)+k^{2}s^{2}\frac{\partial^{2}u}{\partial t^{2}}=f(s,t)$$

Introducing the mode decomposition in this equation, we write:

(37) $$\sum_{i}\left[{\left(s^{4}V_{i}^{\prime \prime }\right)}^{\prime \prime }q_{i}+k^{2}s^{2}V_{i}{\ddot{q}}_{i}\right]=f(s,t)$$

The modes *V*_*i*_ being solutions of Equation (4), the equation can be written as:

(38) $$\sum_{i}s^{2}V_{i}\left[{\ddot{q}}_{i}+\omega_{i}^{2}q_{i}\right]=\frac{1}{k^{2}}f(s,t)$$

Projecting this equation on the spatial mode *V*_*i*_(*s*) and using the orthogonality property, one obtains the dynamic equation for the mode amplitude *q*_*i*_(*t*):

(39) $${\ddot{q}}_{i}+\omega_{i}^{2}q_{i}=\frac{1}{k^{2}}\int_{\varepsilon }^{1}f(s,t)V_{i}(s)ds$$

### Coupling the whisker's rapid dynamics to its quasi-static evolution

The whisker displacement *u*(*s*, *t*) is decomposed in the form *U*(*s*, *t*) = *U*_qs_(*s*, *t*) + *u*(*s*, *t*) where *U*_qs_(*s*, *t*) describes the quasi-static evolution. By introducing this decomposition in Equation (3), one obtains the dynamic equation for *u*(*s*, *t*):

(40) $$\frac{\partial^{2}}{\partial s^{2}}\left(s^{4}\frac{\partial^{2}u}{\partial s^{2}}+s^{4}\frac{\partial^{2}U_{\text{qs}}}{\partial s^{2}}\right)+k^{2}s^{2}\left(\frac{\partial^{2}u}{\partial t^{2}}+\frac{\partial^{2}U_{\text{qs}}}{\partial t^{2}}\right)=0$$

We assume *U*_qs_(*s*, *t*) to obey the small deflection equilibrium Equation (30): (*s*^4^*U*_qs_″)″ = 0. The resonant component of the whisker deflection thus obeys the dynamic equation:

(41) $$\frac{\partial^{2}}{\partial s^{2}}\left(s^{4}\frac{\partial^{2}u}{\partial s^{2}}\right)+k^{2}s^{2}\frac{\partial^{2}u}{\partial t^{2}}=-k^{2}s^{2}{\ddot{U}}_{\text{qs}}(s,t)$$

Using the mode-decomposition *u*(*s*, *t*) = ∑_*i*_*q*_*i*_(*t*)*V*_*i*_(*s*) and the orthogonality of the resonant modes [see Equations (36) and (39)], the amplitude of each mode *q*_*i*_(*t*) is found to obey the dynamic equation:

(42) $${\ddot{q}}_{i}+2\zeta \omega_{i}{\dot{q}}_{i}+\omega_{i}^{2}q=-\int_{\varepsilon }^{1}s^{2}{\ddot{U}}_{\text{qs}}(s,t)V_{i}(s)ds$$

This approach is implemented in the case of a shock. Prior to the shock, the whisker experiences a solid rotation at constant rotation rate γ which simply reads: *U*_qs_(*s*, *t* < 0) = γ(1 − *s*)*t*. Integrating Equation (30) with boundary conditions *U*(1) = 0, U′(1) = γ*t*, *U*(ε) = *U*″(ε) = 0, one obtains the evolution of the quasi-static profile immediately after the shock:

(43) $$U_{\text{qs}}(s,t>0)=\gamma t\frac{(1-s)\left(2s^{2}+\varepsilon^{2}(1+s)-\varepsilon s(3+s)\right)}{2{\left(1-\varepsilon \right)}^{2}s^{2}}$$

Based on these two expressions, one can compute the second time-derivative of the quasi-static profile ${\ddot{U}}_{\text{qs}}(s,t)$:

(44) $${\ddot{U}}_{\text{qs}}(s,t)=\delta (t)\gamma \bar{U}(s)$$

where δ(*t*) is the Dirac function and $\bar{U}(s)$ is a normalized profile that reads:

(45) $$\bar{U}(s)={\dot{U}}_{\text{qs}}(s,0^{+})-{\dot{U}}_{\text{qs}}(s,0^{-})=\frac{\varepsilon {\left(1-s\right)}^{2}(\varepsilon -3s+2s\varepsilon )}{2{\left(1-\varepsilon \right)}^{2}s^{2}}$$

In order to integrate Equation (42), one needs to compute the Green's function *G*(*t*), i.e., the response of the dynamic system, initially at rest, to a Dirac of unit force:

(46) $$\left(\frac{d^{2}}{dt^{2}}+2\zeta \omega_{i}\frac{d}{dt}+\omega_{i}^{2}\right)G(t)=\delta (t)$$

with boundary conditions *G*(*t* < 0) = *G*′(*t* < 0) = 0. For *t* > 0, the general solution reads:

(47) $$G(t>0)=\left(\alpha \cos(\sqrt{1-\zeta^{2}}\omega_{i}t)+\beta \sin(\sqrt{1-\zeta^{2}}\omega_{i}t)\right)e^{-\zeta \omega_{i}t}$$

In order to determine α and β, we integrate Equation (46) between 0^−^ and 0^+^, once and twice:

(48) $$\begin{array}{l} \left(G^{\prime }(0^{+})-G^{\prime }(0^{-})\right)+2\zeta \omega_{i}\left(G(0^{+})-G(0^{-})\right)=1\\ \;\left(G(0^{+})-G(0^{-})\right)=0 \end{array}$$

These two equations and the boundary conditions impose *G*(0^+^) = 0 and *G*′(0^+^) = 1. The solution thus finally reads:

(49) $$\begin{array}{l} \text{For }t>0:G(t)=\frac{1}{\omega_{i}\sqrt{1-\zeta^{2}}}e^{-\zeta \omega_{i}t}\sin\left(\omega_{i}\sqrt{1-\zeta^{2}}t\right)\\ \text{For }t<0:G(t)=0 \end{array}$$

The mode amplitude *q*_*i*_(*t*) of the dynamic component associated with the event occurring at time *t* = 0 simply follows, for *t* > 0:

(50) $$q_{i}(t)=-\left(\int_{\varepsilon }^{1}s^{2}\bar{U}(s)V_{i}(s)ds\right)G(t)$$

### Surgical preparation and whisker stimulation for *in vivo* experiments

Experiments were conducted in conformity with French (JO 2001-464) and European legislation (86/609/CEE) on animal experimentation. An adult male Wistar albino rat (318 g) was anesthetized with urethane (1.5 g/kg, i.p.). Body temperature was maintained at 37°C. The scalp was retracted after a subcutaneous injection of a local anesthetic (Lidocaine 1%). The skull was then cemented to a metal bar. A 1 mm thick metal bar was moved by a linear stepper motor (Linear Motor MLL302, Systro Gmbh) at a speed *V*_bar_ = 60 mm/s. The bar contacted the whisker near its tip. The whiskers were trimmed at the end of the experiment, and their diameters were measured at different positions, from base to tip. This allowed us to determine the length *L* of the ideal (non-truncated) cone.

### High-speed videography of whisker deflections

Whisker movements were recorded at a frame rate of 2.5 kHz with a high-speed camera (Photron Fastcam SA3/105 mm f-2.8 DG Macro Sigma; pixel resolution 55 μm) mounted vertically above the animal. Whiskers were illuminated from below using a backlight (SSLUB, Phlox and PP520, Gardasoft). The camera produces bird's-eye view movies of the whiskers. We choose to study whiskers β and C1 whose curvature plane are essentially horizontal and can thus be entirely imaged with the present optical configuration (Figure 7A).

### Whisker tracking

Detecting the shock-induced deflection wave, whose typical amplitude is of the order of a few tens of microns, required subpixel determination of the whisker centerline profile in each frame. The whisker deflected by the object was tracked using a semi-automated algorithm written in Python. We subtracted the average background image from each frame in the movie. Whisker centerline profiles were then extracted: we scanned all columns of each movie frame in order to extract the pixel of maximal intensity defining the approximate position of the whisker. The precise position of the whisker centerline in each column of the frame was then defined as the barycenter (each pixel is weighted by its intensity) of the pixels surrounding the pixel of maximal intensity (center position ±2 pixel). In order to extract the shock-induced deflection sequence (Figure 7B), the whisker profile determined before the shock was subtracted to each post-shock profile. The deflection sequence was then smoothed using a five pixel wide sliding window. This procedure yields a spatial resolution of ≈1 μ on the shock-induced whisker deflection.

### Analysis of experimental data

Our experimental configuration slightly differed from that considered in the model. In the latter, the whisker rotated at constant speed across a fixed obstacle. In the experiment, the whisker was fixed, and the obstacle was moved linearly at constant speed *V*_bar_. For this shock configuration, it is easy to show that Equation (11) becomes:

(51) $$U(s,t>0)=V_{\text{bar}}t\bar{U}(s)-V_{\text{bar}}\sum_{i}V_{i}(s)\left(\int_{\varepsilon }^{1}s^{2}\bar{U}(s)V_{i}(s)ds\right)\times \frac{e^{-\zeta \omega_{i}t}\sin\left(\sqrt{1-\zeta^{2}}\omega_{i}t\right)}{\sqrt{1-\zeta^{2}}\omega_{i}}$$

The back-illumination geometry combined with the bird's-eye optics results in a shadow region in the vicinity of the object that prevented the tracking of the whisker down to the whisker/object contact point. Similarly, the fur hampered a proper observation of the whisker near the skin. As a consequence, the positions *s* = ε and *s* = 1 could not be determined from the images, and were thus left as free parameters. The wave speed was determined by linear fitting the position of the first deflection minimum as a function of the time elapsed since the first contact (Figure 7C). The four first resonant modes were used to fit the data. Fitting was performed with Matlab's fminsearch function. Correlation between experimental and theoretical data was assessed with Pearson's correlation test on the first 3 and 5 ms for C1 and β whiskers, respectively.

### Conflict of interest statement

The authors declare that the research was conducted in the absence of any commercial or financial relationships that could be construed as a potential conflict of interest.
